# Spectrum of Ductal Carcinoma In Situ (DCIS) Lesions of the Breast: From Morphology to Molecular Characteristics

**DOI:** 10.7759/cureus.69929

**Published:** 2024-09-22

**Authors:** Guttikonda Sathvik, Pavithra V, Leena D Joseph, Chithra Bhanu Challa

**Affiliations:** 1 Department of Pathology, Sri Ramachandra Institute of Higher Education and Research, Chennai, IND

**Keywords:** ar, ductal carcinoma in situ (dcis), egfr, er, her2/neu, ki 67 labelling index., pr

## Abstract

Background

Specific molecular characteristics of invasive breast cancer have been linked to an increased risk of early relapse. Similarly, ductal carcinoma in situ (DCIS) displays a comparable molecular profile, although their prevalence and implications are not yet fully understood.

Aims and objectives

The study design defined a multivariable statistical approach aimed at describing the interplay between the histopathological features of ductal carcinoma in situ (DCIS) and their molecular profile. The objective was to explore the correlations between the histopathological features of DCIS (tumor location, DCIS grade, DCIS type, and presence or absence of comedo necrosis) and various biomarkers like estrogen receptor (ER), progesterone receptor (PR), human epidermal growth factor receptor (HER2/neu), androgen receptor (AR), and epidermal growth factor receptor (EGFR), in addition to the Ki-67 labeling index.

Methods

In this retrospective study, we selected and analyzed 100 diagnosed cases of ductal carcinoma in situ (DCIS) to represent various subtypes, grades, and morphological characteristics. A detailed histopathological review and immunohistopathological staining for ER, PR, HER2/neu, AR, EGFR, and Ki-67 were performed on formalin-fixed paraffin-embedded (FFPE) tumor tissue blocks. Molecular subtyping was done based on the biomarker analysis into luminal A, luminal B, luminal HER2/neu, HER2/neu enriched, and triple-negative subtypes. Statistical analysis was done to examine the correlation between tumor location, histopathological features of ductal carcinoma in situ (DCIS), and the expression of the immunohistochemical markers and the molecular subtypes.

Results

The majority of cases exhibited positivity for estrogen receptor (ER) and progesterone receptor (PR). A strong association was observed between histopathological features (DCIS grade, type, and comedo necrosis) of DCIS and ER/PR status. Additionally, a significant correlation was found between ductal carcinoma in situ (DCIS) grade and HER2/neu status. However, no association was identified between histopathological features and AR or EGFR status. Contrary to expectations, triple-negative DCIS did not show the most aggressive behavior, whereas HER2/neu-positive tumors, particularly high-grade ones, exhibited more aggressive features. No low-grade cases of luminal HER2/neu and HER2/neu-enriched tumors were found. A higher Ki-67 labeling index was found in cases with grade 3 and solid and comedo architectural types of DCIS, while low-grade tumors had a lower Ki-67 labeling index.

Conclusion

These findings suggest that hormone pathways play a crucial role in ductal carcinoma in situ (DCIS) progression, but the molecular interactions are complex and extend beyond simple binary associations. The complex relationship between the histopathological features of ductal carcinoma in situ (DCIS) and its hormone receptor status warrants further investigation with a larger sample size to fully understand the underlying mechanisms. The results challenge the expectation that triple-negative DCIS is the most aggressive subtype, highlighting the need for further research into the prognostic significance of different DCIS subtypes.

## Introduction

Ductal carcinoma in situ (DCIS) is a precursor lesion that can progress to invasive carcinoma, although not all cases will necessarily do so. Notably, DCIS is becoming increasingly prevalent, particularly in the context of breast cancer screening programs, where its detection is on the rise. Several histopathological classification systems have been developed to categorize DCIS into distinct subtypes. The most commonly employed system relies on nuclear morphology, grouping DCIS into high, intermediate, and low nuclear grades. Although this system has some predictive value, with high-grade lesions associated with increased recurrence risk, its clinical utility is hindered by inconsistent reproducibility. In contrast, recent advances in invasive breast cancer research have led to the molecular sub-categorization that prioritizes tumor biology over traditional histopathological analysis, offering a more nuanced understanding of the disease [1].

Diagnosis and grading of DCIS have traditionally been based on histopathological evaluation. These histological assessments assess the architectural patterns and cytomorphological features in tissue biopsies through features like nuclear size, pleomorphism, and presence or absence of comedo-type necrosis to split lesions into low, intermediate, and high grades.

In recent years, invasive breast cancer classification has shifted from solely relying on histomorphology to incorporating molecular characteristics. A study by Perou et al. [2] pioneered this approach by using cDNA microarray analysis to identify distinct breast cancer subtypes based on intrinsic gene expression profiles. Subsequent research by Sorlie et al. [3] refined these subgroups and demonstrated their prognostic significance.

Since then, numerous studies have sought to develop an immunohistochemical panel that can replicate the findings of gene array analysis. Research by Makretsov et al., Nielsen et al., and Matos et al. have laid the groundwork for this effort. A key finding is that a five-biomarker panel consisting of estrogen receptor (ER), progesterone receptor (PR), human epidermal growth factor receptor 2 (Her2), CK5/6, and epidermal growth factor receptor (EGFR) shows promise in categorizing invasive breast cancers into their corresponding molecular subtypes (Cheang et al.). This panel offers a potential surrogate for gene array analysis, enabling more widespread adoption of molecular classification in clinical practice [4-7].

Only a few studies have delved into the molecular subtypes of ductal carcinoma in situ (DCIS), and the existing research presents conflicting findings. Unlike invasive breast cancer, which has seen significant progress in identifying biological subtypes, DCIS remains poorly understood in this regard. The limited research on DCIS subtypes has yielded inconsistent results, highlighting the need for further investigation to clarify the underlying biology of this precursor lesion.

Though it is difficult to ignore the value of these traditional techniques, the field of breast pathology has undergone a dramatic change over the last decade with the application of immunohistochemical (IHC) techniques, which have enabled us, pathologists, to molecularly phenotype the DCIS lesions.

This molecular profiling includes the test for hormone receptors estrogen receptor (ER) and progesterone receptor (PR), human epidermal growth factor receptor 2 (HER2/neu), androgen receptor (AR), epidermal growth factor receptor (EGFR), and proliferation markers like Ki-67. Based on the expression of these biomarkers, DCIS can be molecularly subtyped into luminal A (ER+, PR+, HER2/neu-, Ki-67 labeling index<14%), luminal B (ER+, PR+, HER2/neu-, Ki-67 labeling index>14%), luminal HER2/neu (ER+, PR+/-, HER2/neu+, Ki-67 labeling index-variable), HER2/neu enriched (ER-, PR-, HER2/neu+, Ki-67 variable), and triple-negative (ER-, PR-, HER2/neu-, Ki-67 labeling index-variable) [2].

These biomarkers have revolutionized the understanding of DCIS and may enable treatment strategies to be targeted at the molecular profile of the individual lesions. It creates a synergy of morphological and molecular insights that perhaps have the potential to enhance the predictive performance of models in use for predicting prognosis, hence giving caregivers the ability to separate cases of DCIS with benign tendencies from those with aggressive and probable invasive features. An improved understanding of the molecular makeup of DCIS will allow targeted therapeutic interventions. This will make it possible for clinical treatment strategies to be made more specific by providing a way in which molecular pathways driving the progression of DCIS can be focused by protecting lesions that are at low risk from aggressive treatment; at the same time, those that display high-risk characteristics can be managed aggressively enough. The ultimate goal of combining molecular phenotyping with traditional histomorphology is to refine the approach to DCIS, enable a more intricate and sophisticated understanding of DCIS biology, and consequently allow for optimal patient outcomes through tailored therapeutic interventions. This improved diagnostic and therapeutic framework accounts for the shift to the practice of precision medicine in the treatment of breast cancer, whereby the targeted management program for every DCIS case is supposed to be tailored depending on a distinct molecular signature. The dream of this paradigm shift in the natural history of patients with DCIS is within reach as the field progresses with time and holds a promise that soon breast cancer management will be as unique as the patients themselves.

## Materials and methods

Data collection

This study used a comprehensive approach for the analysis of DCIS lesions by detailed histopathology and immunohistochemical techniques. Hundred representative cases of DCIS were drawn from a large cohort of cases with various surgical excisions, including mastectomy, lumpectomy, and wide local excision. Our selection was purposeful so as to include a wide spectrum of DCIS subtypes, grades, and morphological characteristics that make a comprehensive understanding of the histomorphological heterogeneity. All critical demographic and clinical information, including age at diagnosis, lesion laterality, and tumor location, was collected using the Laboratory Information System (LIS).

Histopathological assessment and molecular subtyping

This histopathological and molecular profiling of each case was central to the core of our methodology. We further evaluated traditional histopathological features, which included the DCIS architectural type and grade and the presence of comedo necrosis. Molecular subtyping, in addition to the histopathological evaluation, was done using immunohistochemical staining consisting of ER (anti-estrogen receptor (SP1) rabbit monoclonal primary antibody), PR (anti-progesterone receptor (1E2) rabbit monoclonal primary antibody), HER2/neu (anti-HER2/neu (4B5) rabbit monoclonal primary antibody), AR (androgen receptor-EP120), EGFR (EGFR-EP22), and Ki-67 (anti-Ki-67 (30-9) rabbit monoclonal primary antibody). This is a retrospective study that was conducted at the Department of Pathology, Sri Ramachandra Medical College and Research Institute, Chennai, between the period of June 2022 and May 2024. We have utilized immunohistochemical staining procedures on formalin-fixed paraffin-embedded (FFPE) samples of surgical resection specimens. We selected paraffin blocks of the cases diagnosed as DCIS by histomorphology for 100 patients. The cases under study included all the DCIS lesions diagnosed in mastectomy, lumpectomy, and wide local excision specimens. The Ventana Benchmark machine was utilized to stain all samples, adhering to stringent guidelines to ensure the accuracy and dependability of the immunohistochemical analysis.

For the immunohistochemical staining interpretation, ER and PR were scored using an Allred scoring system that combines the percentage of positive cells and the intensity of the reaction product in most of the tumors. Only nuclear expression was considered positive [8]. HER2/neu expression was assessed by evaluating the membranous staining in the tumor cells. The amount of staining was then scored based on a scale from 0 to 3+ to determine HER2/neu expression [9]. Any tumor cells that exhibited membrane staining were considered EGFR positive, and any tumor cells that exhibited nuclear staining were considered AR-positive. The percentage of cells that exhibited nuclear staining was measured as the Ki-67 labeling index [10]. The Ki-67 labeling index was stratified into two subcategories: high proliferative tumors with Ki-67 >14% and low proliferative tumors with Ki-67 <14%. Based on the expression of these biomarkers, DCIS was molecularly subtyped into luminal A (ER+, PR+, HER2/neu-, Ki-67 labeling index<14%), luminal B (ER+, PR+, HER2/neu-, Ki-67 labeling index>14%), luminal HER2/neu (ER+, PR+/-, HER2/neu+, Ki-67 labeling index-variable), HER2/neu enriched (ER-, PR-, HER2/neu+, Ki-67 variable), and triple-negative (ER-, PR-, HER2/neu-, Ki-67 labeling index-variable). This has allowed to establish a very analytical approach toward DCIS, combining classical pathological methods with modern molecular diagnostics for a deep understanding of the complexity of the disease.

Statistical analysis

The study proceeded with a statistical analysis with a visual examination of the data, followed by the chi-square and Fisher tests to investigate the correlation between the histopathological features of DCIS with the biomarkers and the molecular subtype. Data processing was done using MS Excel 2024 (Microsoft Corporation, Redmond, Washington, United States), with analysis performed through Microsoft Excel and the free version of Epi Info (version 7.2.5.0). Statistical significance was determined using a p-value of less than 0.05.

## Results

**Figure 1:**
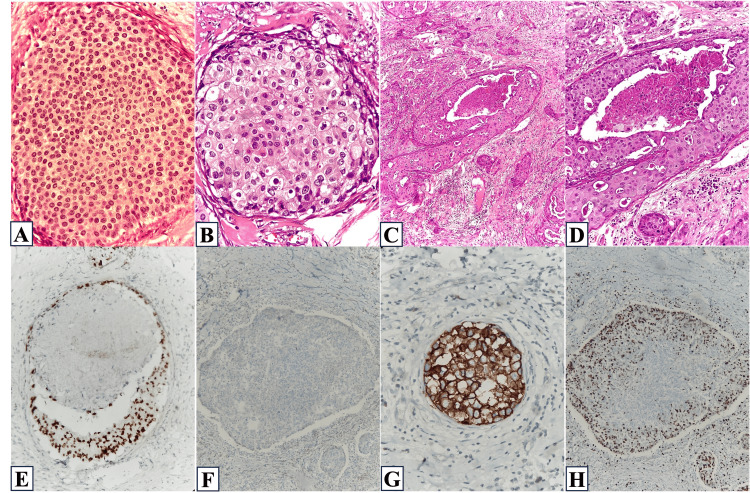
Histological findings A: (H&E, 400x) DCIS grade I: Lesional cells show minimal cellular atypical and pleomorphism. B: (H&E, 400x) DCIS grade II: The lesional cells show moderate nuclear pleomorphism with an intact myoepithelial cell layer. C: (H&E, 100x) DCIS grade 3 with comedo necrosis. D: (H&E, 400x) DCIS grade 3 with comedo necrosis. The lesional cells show marked nuclear atypia and pleomorphism with an intact myoepithelial cell layer. E: (ER IHC, 400x) grade 3 comedo type DCIS showing tumor cells that are positive for ER. F: (PR IHC, 400x) grade 3 solid type DCIS showing tumor cells that are negative for PR. G: (HER2/neu IHC, 400x) grade 3 solid type DCIS showing tumor cells that are positive for PR. H: (Ki-67 IHC, 400x) grade 3 solid type DCIS showing a high Ki-67 labeling index (70%). DCIS: ductal carcinoma in situ; ER: estrogen receptor; PR: progesterone receptor; HER2/neu: human epidermal growth factor receptor; IHC: immunohistochemical

In this study, we examined 100 cases of ductal carcinoma in situ (DCIS) diagnosed between June 2021 and May 2024, with an average age at diagnosis of 56 years (range 32-77). The location of each tumor was macroscopically classified into one of five quadrants: upper outer quadrant (UOQ), upper inner quadrant (UIQ), lower inner quadrant (LIQ), lower outer quadrant (LOQ), and central quadrant. Histopathologically, DCIS was graded into low (grade 1), intermediate (grade 2), and high grades (grade 3). Grade 1 tumors showed a monotonous population of cells showing mild nuclear pleomorphism, finely dispersed chromatin, and occasional nucleoli. Grade 2 tumors showed moderate nuclear pleomorphism and fine chromatin, with a few showing prominent nucleoli. Grade 3 tumors were large (>2.5 cm) and showed marked nuclear pleomorphism, vesicular nuclei, irregular chromatin distribution, and multiple prominent nucleoli. Frequent mitosis was seen in high-grade tumors. Histologically, DCIS was subtyped based on the architectural patterns like solid, cribriform, papillary, and comedo type (Figure 1).

From this core, the statistical analyses proceeded and consisted of visual exploration of the data followed by chi-square and Fisher tests for the identification and evaluation of the relationship of histopathological features and location of DCIS with the immunohistochemical markers. This multifaceted approach, combining the histopathological features with molecular profiling and strong statistical methodologies, places our study at the vanguard for adding important new steps to understanding the biological behaviors and clinical implications of DCIS. The majority of cases (93%) underwent mastectomy, while a few underwent wide local excision (6%) or lumpectomy (1%). Tumors were most commonly located in the upper outer quadrant (43%), followed by the central region (27%), upper inner quadrant (16%), lower inner quadrant (9%), and lower outer quadrant (5%). Histopathological analysis revealed a grade distribution of 14% grade 1, 17% grade 2, and 69% grade 3, with the most common DCIS subtype being comedo (63%), followed by solid (23%), cribriform (8%), and papillary (6%). Comedo necrosis was present in 62 cases (62%) and absent in 38 cases (38%).

Immunohistochemical (IHC) analysis revealed the following biomarker expression profiles in the 100 DCIS cases: estrogen receptor (ER) was positive in 64% and negative in 36% of cases; progesterone receptor (PR) was positive in 60% and negative in 40%; human epidermal growth factor receptor (HER2/neu) was positive in 41% and negative in 59%; androgen receptor (AR) was positive in 77% and negative in 23%; and epidermal growth factor receptor (EGFR) was positive in 36% and negative in 64%. Additionally, 78% of the cases had a Ki-67 labeling index >14%, and 22% of the cases had a Ki-67 labeling index <14%. These IHC findings provide valuable information on the hormone receptor and molecular profiles of these DCIS cases. Molecular subtyping classified the cases into luminal HER2/neu (25%), luminal B (23%), triple-negative (19%), HER2/neu enriched (16%), and luminal A (17%), highlighting the heterogeneity of DCIS. These findings underscore the importance of comprehensive biomarker evaluation for personalized treatment approaches.

A significant statistical association was found between the ER and PR status with histopathological features like DCIS grade, DCIS type, and presence or absence of comedo necrosis (P = 0.046, 0.012, and 0.045, respectively, for ER and 0.016, <0.01, and <0.01, respectively, for PR), while no significant association was found between the ER and PR with the location of the tumor (P >0.05) (Tables 1, 2).

**Table 1:** Correlation of ER expression with DCIS type, DCIS grade, tumor location, and presence or absence of comedo necrosis. DCIS: ductal carcinoma in situ; ER: estrogen receptor; UIQ: upper inner quadrant; LIQ: lower inner quadrant; LOQ: lower outer quadrant; UOQ: upper outer quadrant

		ER Positive (n = 64)	ER Negative (n = 36)	n	p-value	Test
Comedo necrosis	Present	35 (55%)	27 (75%)	62	0.045	Chi-square
	Absent	29 (45%)	9 (25%)	38		
DCIS grade	1	13 (20%)	1 (2.8%)	14	0.046	Chi-square
	2	11 (17%)	6 (17%)	17		
	3	40 (62%)	29 (81%)	69		
DCIS type	Comedo	33 (52%)	30 (83%)	63	0.012	Fisher
	Solid	20 (31%)	3 (8.3%)	23		
	Cribriform	6 (9.4%)	2 (5.6%)	8		
	Papillary	5 (7.8%)	1 (2.8%)	6		
Tumor location	UOQ	26 (41%)	17 (47%)	43	0.51	Fisher
	Central	18 (28%)	9 (25%)	27		
	UIQ	9 (14%)	7 (19%)	16		
	LIQ	6 (9.4%)	3 (8.3%)	9		
	LOQ	5 (7.8%)	0 (0%)	5		

**Table 2:** Correlation of PR expression with DCIS type, DCIS grade, tumor location, and presence or absence of comedo necrosis. DCIS: ductal carcinoma in situ; PR: progesterone receptor; UIQ: upper inner quadrant; LIQ: lower inner quadrant; LOQ: lower outer quadrant; UOQ: upper outer quadrant

		PR Positive (n = 60)	PR Negative (n = 40)	n	p-value	Test
Comedo necrosis	Present	31 (52%)	31 (78%)	62	<0.01	Chi-square
	Absent	29 (48%)	9 (22%)	38		
DCIS grade	1	13 (22%)	1 (2.5%)	14	0.016	Chi-square
	2	11 (18%)	6 (15%)	17		
	3	36 (60%)	33 (82%)	69		
DCIS type	Comedo	30 (50%)	33 (82%)	63	<0.01	Fisher
	Solid	19 (32%)	4 (10%)	23		
	Cribriform	6 (10%)	2 (5%)	8		
	Papillary	5 (8.3%)	1 (2.5%)	6		
Tumor location	UOQ	24 (40%)	19 (48%)	43	0.36	Fisher
	Central	17 (28%)	10 (25%)	27		
	UIQ	8 (13%)	8 (20%)	16		
	LIQ	6 (10%)	3 (7.5%)	9		
	LOQ	5 (8.3%)	0 (0%)	5		

A significant statistical association was found between HER2/neu and DCIS grade (P <0.001), while no significant association was found between HER2/neu with other histopathological features like DCIS type, tumor location, and presence or absence of comedo necrosis (P >0.05) (Table 3).

**Table 3:** Correlation of HER2/neu expression with DCIS type, DCIS grade, tumor location, and presence or absence of comedo necrosis. DCIS: ductal carcinoma in situ; HER2/neu: human epidermal growth factor receptor; UIQ: upper inner quadrant; LIQ: lower inner quadrant; LOQ: lower outer quadrant; UOQ: upper outer quadrant

		HER2/neu Negative (n = 59)	HER2/neu Positive (n = 41)	n	p-value	Test
Comedo necrosis	Present	32 (54%)	30 (73%)	62	0.055	Chi-square
	Absent	27 (46%)	11 (27%)	38		
DCIS grade	1	14 (24%)	0 (0%)	14	<0.001	Chi-square
	2	12 (20%)	5 (12%)	17		
	3	33 (56%)	36 (88%)	69		
DCIS type	Comedo	32 (54%)	31 (76%)	63	0.2	Fisher
	Solid	16 (27%)	7 (17%)	23		
	Cribriform	6 (10%)	2 (4.9%)	8		
	Papillary	5 (8.5%)	1 (2.4%)	6		
Tumor location	UOQ	29 (49%)	14 (34%)	43	0.49	Fisher
	Central	16 (27%)	11 (27%)	27		
	UIQ	8 (14%)	8 (20%)	16		
	LIQ	4 (6.8%)	5 (12%)	9		
	LOQ	2 (3.4%)	3 (7.3%)	5		

There was no significant statistical correlation between AR and EGFR expression and various histopathological features of DCIS, such as grade, type, location, and comedo necrosis status, as evidenced by P >0.05 (Tables 4, 5).

**Table 4:** Correlation of AR expression with DCIS type, DCIS grade, tumor location, and presence or absence of comedo necrosis. AR: androgen receptor; DCIS: ductal carcinoma in situ; UIQ: upper inner quadrant; LIQ: lower inner quadrant; LOQ: lower outer quadrant; UOQ: upper outer quadrant

		AR Positive (n = 77)	AR Negative (n = 23)	n	p-value	Test
Comedo necrosis	Present	49 (64%)	13 (57%)	62	0.54	Chi-square
	Absent	28 (36%)	10 (43%)	38		
DCIS grade	1	12 (16%)	2 (8.7%)	14	0.77	Fisher
	2	13 (17%)	4 (17%)	17		
	3	52 (68%)	17 (74%)	69		
DCIS type	Comedo	47 (61%)	16 (70%)	63	0.37	Fisher
	Solid	19 (25%)	4 (17%)	23		
	Cribriform	5 (6.5%)	3 (13%)	8		
	Papillary	6 (7.8%)	0 (0%)	6		
Tumor location	UOQ	32 (42%)	11 (48%)	43	0.39	Fisher
	Central	23 (30%)	4 (17%)	27		
	UIQ	10 (13%)	6 (26%)	16		
	LIQ	7 (9.1%)	2 (8.7%)	9		
	LOQ	5 (6.5%)	0 (0%)	5		

**Table 5:** Correlation of EGFR expression with DCIS type, DCIS grade, tumor location, and presence or absence of comedo necrosis. EGFR: epidermal growth factor receptor; DCIS: ductal carcinoma in situ; UIQ: upper inner quadrant; LIQ: lower inner quadrant; LOQ: lower outer quadrant; UOQ: upper outer quadrant

		EGFR Negative (n = 64)	EGFR Positive (n = 36)	n	p-value	Test
Comedo necrosis	Present	38 (59%)	24 (67%)	62	0.47	Chi-square
	Absent	26 (41%)	12 (33%)	38		
DCIS grade	1	10 (16%)	4 (11%)	14	0.77	Chi-square
	2	10 (16%)	7 (19%)	17		
	3	44 (69%)	25 (69%)	69		
DCIS type	Comedo	37 (58%)	26 (72%)	63	0.54	Fisher
	Solid	16 (25%)	7 (19%)	23		
	Cribriform	6 (9.4%)	2 (5.6%)	8		
	Papillary	5 (7.8%)	1 (2.8%)	6		
Tumor location	UOQ	33 (52%)	10 (28%)	43	0.081	Fisher
	Central	14 (22%)	13 (36%)	27		
	UIQ	7 (11%)	9 (25%)	16		
	LIQ	6 (9.4%)	3 (8.3%)	9		
	LOQ	4 (6.2%)	1 (2.8%)	5		

A significant statistical association was observed between DCIS grade and type and the Ki-67 labeling index (P <0.001 and <0.01, respectively). In contrast, no significant associations were found between Ki-67 labeling index and tumor location and the presence or absence of comedo necrosis (P >0.05) (Table 6).

**Table 6:** Correlation of Ki-67 labelling index with DCIS type, DCIS grade, tumor location, and presence or absence of comedo necrosis. DCIS: ductal carcinoma in situ; UIQ: upper inner quadrant; LIQ: lower inner quadrant; LOQ: lower outer quadrant; UOQ: upper outer quadrant

		Ki-67 Lebelling index >14% (n = 78)	Ki-67 Lebelling index <14% (n = 22)	n	p-value	Test
Comedo necrosis	Present	50 (64%)	12 (55%)	62	0.41	Chi-square
	Absent	28 (36%)	10 (45%)	38		
DCIS grade	1	5 (6.4%)	9 (41%)	14	<0.001	Fisher
	2	12 (15%)	5 (23%)	17		
	3	61 (78%)	8 (36%)	69		
DCIS type	Comedo	56 (72%)	7 (32%)	63	<0.01	Fisher
	Solid	14 (18%)	9 (41%)	23		
	Cribriform	4 (5.1%)	4 (18%)	8		
	Papillary	4 (5.1%)	2 (9.1%)	6		
Tumor location	UOQ	34 (44%)	9 (41%)	43	0.059	Fisher
	Central	19 (24%)	8 (36%)	27		
	UIQ	16 (21%)	0 (0%)	16		
	LIQ	6 (7.7%)	3 (14%)	9		
	LOQ	3 (3.8%)	2 (9.1%)	5		

A statistically significant association was observed between DCIS grade and type across all molecular subtypes of DCIS (P <0.01 for both DCIS grade and type). In contrast, no significant associations were found between tumor location or presence/absence of comedo necrosis and molecular subtypes of DCIS (P >0.05) (Table 7).

**Table 7:** Correlation of molecular subtype with DCIS type, DCIS grade, tumor location, and presence or absence of comedo necrosis. DCIS: ductal carcinoma in situ; UIQ: upper inner quadrant; LIQ: lower inner quadrant; LOQ: lower outer quadrant; UOQ: upper outer quadrant

		Luminal HER2/neu (n = 25)	Luminal B (n = 23)	Triple-Negative (n = 19)	Luminal A (n = 17)	HER2/neu enriched (n = 16)	n	p- value	Test
Comedo necrosis	Present	17 (68%)	11 (48%)	14 (74%)	8 (47%)	12 (75%)	62	0.18	Chi-square
	Absent	8 (32%)	12 (52%)	5 (26%)	9 (53%)	4 (25%)	38		
DCIS grade	1	0 (0%)	4 (17%)	1 (5.3%)	9 (53%)	0 (0%)	14	<0.001	Fisher
	2	3 (12%)	5 (22%)	3 (16%)	4 (24%)	2 (12%)	17		
	3	22 (88%)	14 (61%)	15 (79%)	4 (24%)	14 (88%)	69		
DCIS type	Comedo	18 (72%)	12 (52%)	16 (84%)	4 (24%)	13 (81%)	63	<0.01	Fisher
	Solid	5 (20%)	7 (30%)	1 (5.3%)	8 (47%)	2 (12%)	23		
	Cribriform	2 (8%)	1 (4.3%)	2 (11%)	3 (18%)	0 (0%)	8		
	Papillary	0 (0%)	3 (13%)	0 (0%)	2 (12%)	1 (6.2%)	6		
Tumor location	UOQ	8 (32%)	12 (52%)	10 (53%)	6 (35%)	7 (44%)	43	0.38	Fisher
	Central	7 (28%)	4 (17%)	5 (26%)	8 (47%)	3 (19%)	27		
	UIQ	4 (16%)	5 (22%)	3 (16%)	0 (0%)	4 (25%)	16		
	LIQ	3 (12%)	2 (8.7%)	1 (5.3%)	1 (5.9%)	2 (12%)	9		
	LOQ	3 (12%)	0 (0%)	0 (0%)	2 (12%)	0 (0%)	5		

## Discussion

This study sheds light on the complex molecular landscape of ductal carcinoma in situ (DCIS). The majority of cases (64% ER-positive and 60% PR-positive) were hormone receptor-positive tumors, predominantly of high grade (grade 3) [11,12]. Notably, most ER and PR-positive tumors were grade 3 and exhibited comedo necrosis, contradicting previous findings by Barnes et al. and Yang et al. that associated hormone receptor-positive tumors with lower grade and less comedo necrosis [13,14]. Consistent with previous studies by Yang et al. and Akrida et al., most HER2/neu positive tumors (88%) were high grade (grade 3) [14,15]. A positive correlation was observed between the Ki-67 labeling index and DCIS grade, with higher indices in high-grade and aggressive subtypes (solid and comedo architectural types) [16]. Interestingly, most luminal HER2/neu (88%), HER2/neu enriched (72%), and triple-negative (79%) DCIS cases were high grade, whereas only 24% of luminal A tumors were of grade 3. Around 53% of luminal A tumors were low-grade DCIS, while none of the luminal HER2/neu and HER2/neu-enriched types and only 5.3% of triple-negative DCIS were of grade 1. Contrary to expectations, triple-negative tumors did not exhibit the most aggressive morphology but instead showed lower histological grades than HER2/neu-positive tumors [17]. The majority of triple-negative (84%), luminal HER2/neu (72), and HER2/neu enriched (81%) cases showed comedo-type architectural patterns, while luminal A was predominantly solid type (47%) [18]. These findings warrant further investigation to elucidate the complex interactions between these biomarkers and DCIS.

The discovery of these biomarkers has transformed our understanding of ductal carcinoma in situ (DCIS), enabling targeted therapeutic interventions based on the molecular pathways driving DCIS progression and tailored to individual tumor profiles. By integrating the histomorphology and molecular profile, clinicians can enhance the prognostic accuracy, distinguish low-risk from more aggressive high-risk DCIS cases, and achieve optimal patient outcomes. Targeted studies are necessary to unravel the molecular mechanisms underlying DCIS progression and inform the development of personalized treatment strategies.

This study, however, has a few limitations. Firstly, the sample size of 100 cases may not be representative of all the DCIS cases, and a larger cohort would provide more robust findings. Furthermore, the study lacked long-term follow-up data, which would be essential to understanding the impact of molecular subtypes on treatment outcomes and disease progression. Lastly, the study was conducted at a single institution, which may not be generalizable to other populations or institutions.

## Conclusions

In conclusion, our study provides a comprehensive molecular sub-characterization of ductal carcinoma in situ (DCIS), revealing a complex landscape of hormone receptor expression, including estrogen receptor (ER), progesterone receptor (PR), as well as human epidermal growth factor receptor (HER2/neu) expression. We found that the majority of DCIS cases were hormone receptor-positive (ER and PR), with most ER and PR-positive tumors exhibiting high-grade features and comedo necrosis. Contrary to expectations, triple-negative DCIS did not display the most aggressive behavior, instead showing lower grades suggestive of a relatively indolent behavior compared to HER2/neu-positive tumors. Our findings also highlighted distinct molecular subtype-specific differences in grade, comedo necrosis, and Ki-67 labeling index. These results have important implications for the management and treatment of DCIS, suggesting that molecular subtyping should be considered in clinical decision-making to optimize patient outcomes in all the cases diagnosed with ductal carcinoma in situ (DCIS). A larger sample size and more detailed molecular profiling will be at the core of future studies, so that the molecular profile of DCIS biology can be illustrated in greater detail, thus aiming to advance the diagnostic, prognostic, and therapeutic approaches to this multifaceted disease.
